# Quick Estimation Model for the Concentration of Indoor Airborne Culturable Bacteria: An Application of Machine Learning

**DOI:** 10.3390/ijerph14080857

**Published:** 2017-07-30

**Authors:** Zhijian Liu, Hao Li, Guoqing Cao

**Affiliations:** 1 Department of Power Engineering, School of Energy, Power and Mechanical Engineering, North China Electric Power University, Baoding 071003, China; zhijianliu@ncepu.edu.cn; 2 Department of Chemistry, The University of Texas at Austin, 105 E. 24th Street, Stop A5300, Austin, TX 78712, USA; 3 Institute for Computational and Engineering Sciences, The University of Texas at Austin, 105 E. 24th Street, Stop A5300, Austin, TX 78712, USA; 4 Institute of Building Environment and Energy, China Academy of Building Research, Beijing 100013, China

**Keywords:** indoor airborne culturable bacteria, PM_2.5_ and PM_10_, estimation model, machine learning, artificial neural network

## Abstract

Indoor airborne culturable bacteria are sometimes harmful to human health. Therefore, a quick estimation of their concentration is particularly necessary. However, measuring the indoor microorganism concentration (e.g., bacteria) usually requires a large amount of time, economic cost, and manpower. In this paper, we aim to provide a quick solution: using knowledge-based machine learning to provide quick estimation of the concentration of indoor airborne culturable bacteria only with the inputs of several measurable indoor environmental indicators, including: indoor particulate matter (PM_2.5_ and PM_10_), temperature, relative humidity, and CO_2_ concentration. Our results show that a general regression neural network (GRNN) model can sufficiently provide a quick and decent estimation based on the model training and testing using an experimental database with 249 data groups.

## 1. Introduction

Many indoor microorganisms are potential threats to human health [1,2,3,4,5,6]. High concentrations of airborne bacteria or fungi may lead to some common diseases, such as respiratory symptoms, allergies, and asthma [7,8,9]. Studies have shown that outdoor particulate matter (PM) air pollution could cause formidable public health risk due to inhalable microorganisms that exist in PM_2.5_ and PM_10_ pollutants [10]. Our recent studies further found that relatively high concentrations of indoor PM_2.5_ and PM_10_ pollutants are qualitatively related to high concentrations of indoor inhalable bacteria and fungi [8,11], which seriously jeopardizes human health.

To reduce the potential threats of indoor inhalable microorganisms, a common method is the use of germicides [12] or antifungal agents [13]. However, before deciding whether to use these products, people usually struggle with a simpler question: what are the approximate concentrations of bacteria and fungi in my house? Experimentally, the measurements of bacterial or fungal concentrations are complicated, including sampling and culture, which require high economic cost and a long timescale. To estimate the indoor fungal concentration, we previously found a linear relationship between PM and indoor fungal concentration [8], which is applicable for indoor fungi estimation. However, to our best knowledge, very few reports have shown an effective estimation method for the concentration of indoor airborne culturable bacteria.

To estimate the concentration of indoor airborne culturable bacteria, a good first step is to make full use of the indoor environmental indicators. With the assumption that some important indoor indicators (e.g., PM, temperature, relative humidity and CO_2_ concentration) correlate (or partially correlate) with the growth of bacteria, they can be used as the independent variables for the prediction of bacterial concentration. Setting the bacterial concentration as the dependent variable, we can further use predictive machine learning methods trained from the experimental database. A good machine learning model can effectively “learn” from the existing database and perform precise and robust predictions. With the algorithm developments made during the past few decades, there are currently a large number of machine learning methods that can be performed for numerical regression, such as artificial neural network (ANN) [14] and support vector machine (SVM) [15]. Artificial neural network (ANN) is a proven powerful machine learning tool for numerical prediction [16,17], classification [18], and pattern recognition [19]. During recent years, it has been widely used in chemical [20,21], biological [22], medical [23], environmental [24], and engineering [17,25,26] applications.

In this communication, we aim to propose a quick estimation method of the concentration of indoor airborne culturable bacteria using ANN models. Our results show that with the simple inputs of indoor PM_2.5_ and PM_10_, temperature, relative humidity, and CO_2_ concentration, the model trained from our experimental database with 249 data groups can effectively predict the concentration of indoor airborne culturable bacteria with relatively low root mean square errors (RMS errors).

## 2. Modeling Methods 

The structure of a typical ANN consists of three different types of layers, including the input, hidden, and output layers. Each layer consists of a certain number of neurons. Each neuron in the input layer represents an independent variable, while the neurons in the output layer are the dependent variables. Each neuron interconnects with all the neurons in the adjacent layer(s). The training of an ANN is essentially the searching of optimal weights between all the pairs of neurons. Currently, there are a large number of ANN algorithms, such as back-propagation neural network (BPNN) [27,28,29], general regression neural network (GRNN) [17,22], and extreme learning machine (ELM) [30,31,32]. Though they have different weight calculation strategies, the basic principles and structures are similar. The introduction of a general schematic ANN structure can be seen in Reference [33]. In this study, we mainly used a GRNN as the ANN model for training. Compared to conventional ANN methods, GRNN is a state-of-the-art algorithm that has the advantages of fast training and fixed structure [17]. People do not need to compare varying numbers of neurons in the hidden layer during the training and testing, which saves time and computational cost. Details of the principle of a typical GRNN can be found in the work conducted by Specht [34].

To develop a model for estimating the concentration of indoor airborne culturable bacteria, we chose a series of independent variables that can be easily measured inside a building, including: (i) indoor PM_2.5_, (ii) indoor PM_10_, (iii) temperature, (iv) relative humidity, and (v) CO_2_ concentration. All the data were measured in various buildings in Baoding, one of the cities that recently suffered from the most serious PM_2.5_ pollutions in China. Descriptive statistics of the 249 measured data groups are shown in Table 1, which shows that the data ranges of all our measured variables are wide enough for a machine learning model training. Details on the measurements have been mentioned in our previous articles [8,11,35]. With the assumption that all these factors are correlated (or partially correlated) to the concentration of indoor airborne bacteria, these independent variables were considered as the inputs of the GRNN for model training. The measured bacterial concentration was then assigned as the output of the GRNN. Statistical analysis of the potential relationships between the independent variables and the dependent variable are shown in Section 3.1. For each training and testing process, the dataset was divided into two different subsets, the training and testing sets. Training sets were used for the data training, from which the GRNN model can “learn” via a “black-box” fitting process; testing sets were used for the testing of the precision of the trained GRNN models. In our study, we used different percentages of training and testing sets for model developments and compared the precision and robustness of each model. The training and testing of each percentage were repeated 200 times. RMS errors were calculated from the testing results for comparison. Details on the training and testing results are shown in Section 3.2. For comparison, we also performed training and testing processes on a multilayer feedforward neural network (MLFN) [36] with varying numbers of hidden nodes (2–15) in a single hidden layer. The number of trials for each MLFN training was set as 100,000. All the modeling works were performed on a laptop (ThinkPad series, Lenovo, Morrisville, NC, USA). 

## 3. Results and Discussion

### 3.1. Statistical Analysis

**Figure 1:**
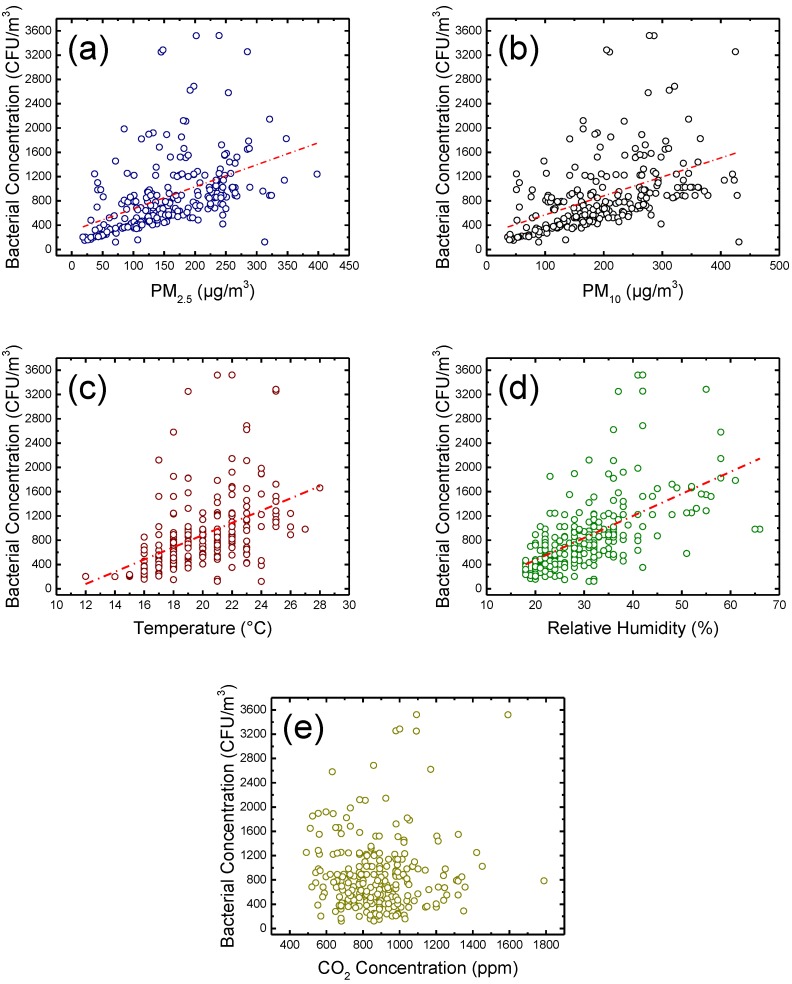
Relationships between the concentration of indoor airborne culturable bacteria vs. (**a**) indoor PM_2.5_, (**b**) indoor PM_10_, (**c**) temperature, (**d**) relative humidity, and (**e**) CO_2_ concentration.

Figure 1 shows the potential relationships between the measured bacterial concentration and the measured independent variables. It can be seen that the indoor PM_2.5_ and PM_10_, temperature, and relative humidity correlate moderately with the concentration of indoor airborne culturable bacteria. However, in terms of CO_2_ concentration, the correlation is insignificant. Interestingly, the concentrations of indoor PM_2.5_ and PM_10_ have less correlation with the bacterial concentration than with the indoor fungal concentration reported in a previous study [8], which indicates that the bacterial concentration is less dependent than the fungal concentration on these environmental indicators. This can also probably explain why there are always some data points that significantly deviate from the regression lines in Figure 1. Also, the correlation between relative humidity and the concentration of indoor airborne culturable bacteria is consistent with the previous studies [37]. All these results show that we cannot use a simple linear regression model with a single independent variable to predict the bacterial concentration, because the growth of bacterium usually depends on more than one environmental factor. Therefore, to more precisely predict the bacterial concentration, a non-linear fitting technique is necessary. 

### 3.2. Machine Learning-Based Prediction

**Figure 2:**
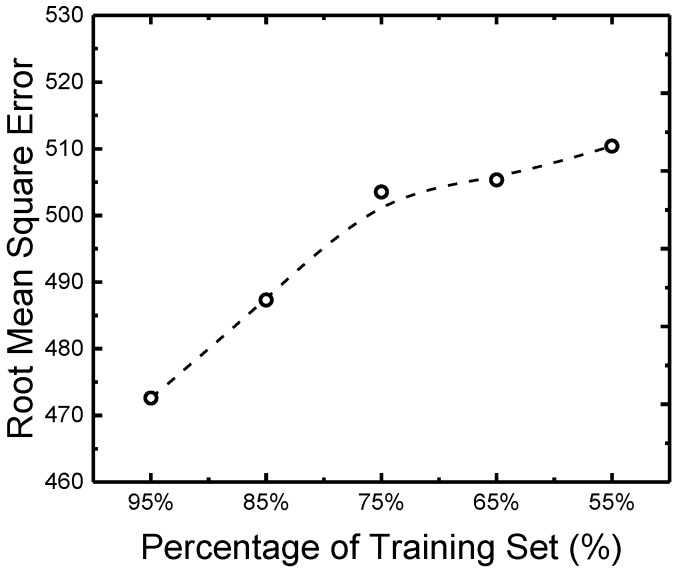
Average root mean square errors (RMS errors) of the testing processes with the training sets percentage varying from 55% to 95%. The training with each percentage was repeated 200 times.

During the development of the GRNN, the training sets of 95%, 85%, 75%, 65%, and 55%, were respectively used. The average RMS errors (acquired from 200 repeated training and testing processes) are shown in Figure 2. It can be clearly seen that with a higher percentage of training set, the average RMS error in the testing process becomes lower. This is consistent with the principle of a non-linear fitting process: training with a larger dataset will lead to a lower risk of over-fitting. Due to the high economic cost for the bacterial concentration measurement in our study, we so far could only acquire 249 data groups. Though this database is large enough for a promising model training, for practical applications, it is always recommended that using a larger experimental database can lead to better predictive performance. 

Meanwhile, the performance of the GRNN was also compared with the multiple linear regression and MLFN models with varying numbers of hidden nodes (Table 2). The RMS errors (calculated from the testing set) shown in Table 2 were extracted from 15 repeated model training processes. It can be seen that the average RMS error of the GRNN is significantly lower than those of other models we developed. Though its minimum RMS error is higher than some other models, the range between the maximum and minimum RMS error of the GRNN is the smallest among all the models we developed. These results show that the GRNN not only has generally low average RMS error, but also has very good stability. For this reason, we conclude that with the training using our experimental database (Table 1), the GRNN outperforms the MLFN and multiple linear regression models. 

**Figure 3:**
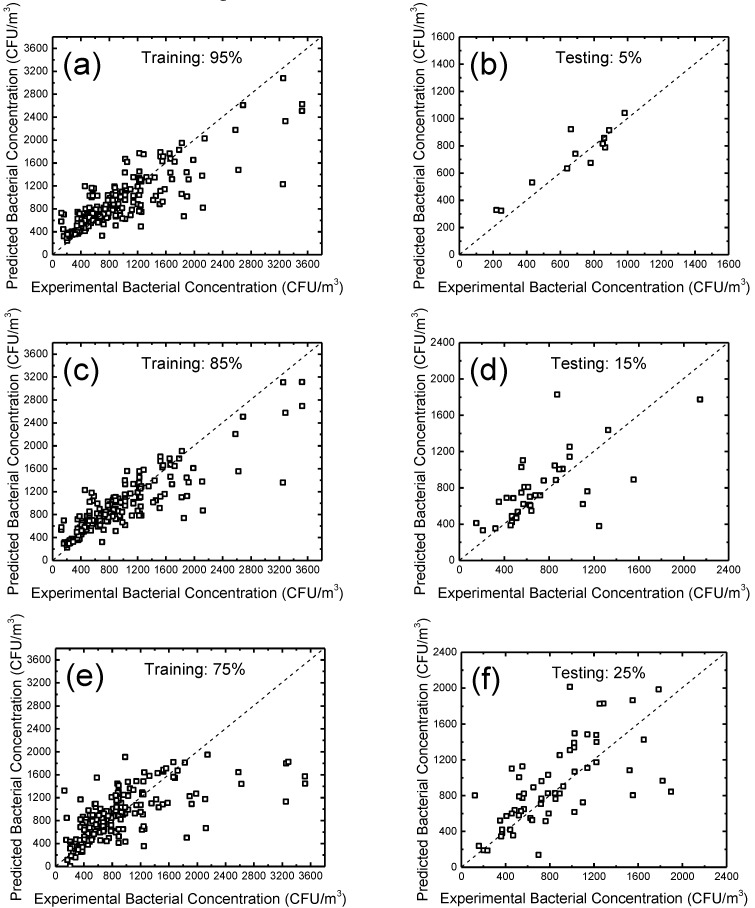
Typical comparative results of predicted bacterial concentration vs. experimental bacterial concentration in various percentages of training and testing sets. (**a**,**b**) Training and testing results with the training and testing percentages of 95% and 5%, respectively. (**c**,**d**) Training and testing results with the training and testing percentages of 85% and 15%, respectively. (**e**,**f**) Training and testing results with the training and testing percentages of 75% and 25%, respectively. Diagonals represent the function of *y* = *x*.

To show the training and testing results of the GRNN more clearly, we picked three typical modeling results for discussions, as shown in Figure 3 (a and b: training and testing sets of 95% and 5%, respectively; c and d: training and testing sets of 85% and 15%, respectively; e and f: training and testing sets of 75% and 25%, respectively). It can be seen that with relatively high percentages of training set (e.g., 95% in Figure 3a and 85% in Figure 3c), the training results look quite decent (except for several points that deviate from the diagonals), leading to good testing results, as shown in Figure 3b,d, respectively. A plausible explanation for the discrete points is that the data groups with relatively high bacterial concentration are not enough to ensure a good fitting in a high concentration area. With our experimental conditions, it was found that the average bacterial concentration was around 877.3 colony forming units per m^3^ (CFU/m^3^) (Table 1), which was much lower than the maximum we have observed (3522 CFU/m^3^, Table 1). We expect that, with a larger database in practical applications, this problem can be addressed properly. In terms of the training set with a relatively low percentage (e.g., a training set of 75%, as shown in Figure 3e), the training fails to precisely fit the data groups with a relatively large bacterial concentration, leading to weak prediction performance in the testing set (Figure 3f). We also found that using the percentages of the training set lower than 75% resulted in even worse predictive performance in their corresponding testing sets. These results, together with Figure 2, show that with a relatively large training set, the developed GRNN method is a promising ANN model for predicting the concentration of indoor airborne culturable bacteria with a few inputs of measurable indoor environmental indicators.

**Figure 4:**
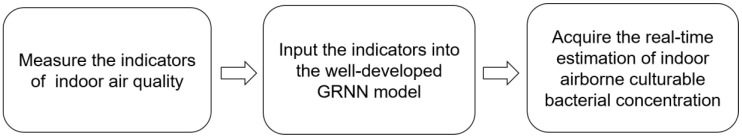
A proposed framework for the real-time estimation of the concentration of indoor airborne culturable bacteria.

A good potential application of such a model is the real-time measurement of the concentration of indoor airborne culturable bacteria. Because all the independent variables used in this model can be acquired in a short period, the real-time bacterial concentration can also be evaluated once the values of the independent variables are inputted into the GRNN model. The flow chart of the proposed measurement method is shown in Figure 4. We expect that this quick evaluation method can dramatically shorten the measurement of bacterial concentration from days to seconds. In future study, we will continue to develop a user-friendly software that can be applicable for real measurements.

## 4. Conclusions

In this communication, we have shown that a machine learning-based method can perform fast estimation of the concentration of indoor airborne culturable bacteria. We found that with the inputs of some indoor environmental indicators that can be easily measured (indoor PM_2.5_ and PM_10_, indoor temperature, relative humidity, and CO_2_ concentration), a well-trained GRNN model can help to quickly acquire the estimated concentration. This novel estimation can dramatically reduce the measurement time from days to seconds, saving much time, economic cost, and manpower. We expect that this estimation method can also be applied to the quick measurement of indoor fungal and virus concentrations.

## Figures and Tables

**Table 1:** Descriptive statistics of variables for the 249 measured data groups.

Item	Indoor PM_2.5_ (μg/m^3^)	Indoor PM_10_ (μg/m^3^)	Temperature (°C)	Relative Humidity (%)	CO_2_ Concentration (ppm)	Bacterial Concentration (CFU/m^3^)
Maximum	398	431	28	66	1789	3522
Minimum	18	36	12	18	491	122
Range	380	395	16	48	1298	3400
Average	157	198	20	31	881	877.3
Standard Deviation	76	91	3	10	204	596.6

Note: PM: particulate matter; CFU: colony forming units.

**Table 2:** RMS errors of different predictive models. Multilayer feedforward neural networks (MLFNs) with different numbers of hidden neurons are represented as MLFN-*x*, where *x* represents the number of hidden neurons. The average RMS error of each model was acquired from 15 repeated training and testing processes.

Model	Average RMS Error (Testing)	Maximum RMS Error (Testing)	Minimum RMS Error (Testing)	Range
Linear Regression	488.51	742.40	183.63	558.77
GRNN	412.69	630.87	243.59	387.28
MLFN-*2*	501.51	842.10	191.61	650.49
MLFN-*3*	567.56	936.49	261.92	674.57
MLFN-*4*	551.14	762.22	238.96	523.26
MLFN-*5*	537.75	905.98	221.88	684.09
MLFN-*6*	618.37	1168.41	169.72	998.69
MLFN-*7*	613.02	1232.63	137.23	1095.40
MLFN-*8*	554.64	852.59	239.91	612.68
MLFN-*9*	617.65	873.16	198.35	674.81
MLFN-*10*	646.42	924.84	255.50	669.34
MLFN-*11*	614.87	1015.63	218.95	796.68
MLFN-*12*	644.37	1313.63	223.55	1090.08
MLFN-*13*	675.69	1033.43	279.78	753.65
MLFN-*14*	746.24	1304.57	321.40	983.17
MLFN-*15*	716.42	1761.50	250.64	1510.86
